# Predictors of Recurrence After Surgical Evacuation of Chronic Subdural Hematoma: A Single-Center Retrospective Study of 250 Patients With Development of a Simple Clinical Score

**DOI:** 10.7759/cureus.106834

**Published:** 2026-04-11

**Authors:** Hamza Sriri, Mehdi Mdarhri, Oualid Hmamouche, Marouane Hammoud, Faycal Lakhdar, Mohammed Benzagmout, Chakour Khalid, Mohammed Chaouielfaiz

**Affiliations:** 1 Neurosurgery, Hassan II University Hospital, Fez, MAR

**Keywords:** anticoagulants, chronic subdural hematoma, pneumocephalus, predictive score, recurrence, risk factors, septations

## Abstract

Background: Chronic subdural hematoma (CSDH) is a common neurosurgical condition with a high recurrence rate. Identifying reliable predictors of recurrence is essential to improve postoperative management. However, data from low-resource settings remain limited, and few studies provide clinically applicable predictive tools.

Objective: This study aimed to identify independent predictors of recurrence after surgical evacuation of CSDH and to develop a simple, clinically applicable predictive score.

Methods: We conducted a retrospective, single-center study including 250 patients who underwent surgery for CSDH at Centre Hospitalier Universitaire Hassan II, Fes. Clinical, radiological, and surgical variables were analyzed. Univariate and multivariate analyses were performed to identify independent predictors of recurrence. A predictive score was developed based on significant variables.

Results: The recurrence rate was 20.4%. Multivariate analysis identified anticoagulant therapy, presence of septations, and postoperative pneumocephalus as independent predictors of recurrence. A simple predictive score ranging from 0 to 3 was developed. Recurrence rates increased progressively with higher scores.

Conclusion: This study confirms key predictors of CSDH recurrence and proposes a simple, clinically applicable scoring system to stratify patients according to recurrence risk. This tool may help guide postoperative monitoring and improve clinical decision-making. Further validation in multicenter studies is required.

## Introduction

Chronic subdural hematoma (CSDH) is one of the most common neurosurgical conditions, particularly in the elderly population. Its incidence has significantly increased over recent decades due to population aging and the widespread use of anticoagulant and antiplatelet therapies. This growing prevalence represents an important public health concern in neurosurgical practice [1,2].

Clinically, CSDH presents with a wide spectrum of symptoms ranging from mild headaches to severe neurological deficits, reflecting the progressive accumulation of blood in the subdural space. The diagnosis is primarily based on computed tomography (CT), which provides essential information regarding hematoma characteristics such as density, volume, and mass effect, and plays a key role in therapeutic decision-making [3].

Surgical evacuation, most commonly via burr-hole drainage, remains the standard treatment for symptomatic cases and is associated with favorable outcomes in most patients. However, recurrence remains a major challenge, occurring in approximately 5% to 30% of cases, often requiring reoperation and increasing morbidity and healthcare costs [2,4].

Several factors have been proposed as predictors of recurrence, including patient-related variables such as age and anticoagulant therapy, radiological features such as bilateral hematomas, mixed density, and internal septations, as well as surgical factors including the use of drainage and the presence of postoperative pneumocephalus. These radiological features, namely multiloculated hematomas with septations, mixed-density hematomas, bilateral hematomas, and postoperative pneumocephalus, are particularly relevant in assessing recurrence risk [5-8].

Despite these findings, the relative contribution of these factors remains controversial, and their integration into simple and clinically applicable predictive tools is still limited. Moreover, most available data originate from high-resource settings, with limited evidence from low- and middle-income countries [9].

The aim of this study was to identify independent predictors of recurrence after surgical evacuation of CSDH in a cohort of 250 patients and to develop a simple, clinically applicable predictive score to facilitate risk stratification and improve postoperative management.

## Materials and methods

Study design and setting

We conducted a retrospective, single-center study in the Department of Neurosurgery at Centre Hospitalier Universitaire Hassan II, Fes, Morocco, including patients treated for chronic subdural hematoma (CSDH) between January 2020 and August 2025.

Study population

All patients diagnosed with CSDH and managed surgically during the study period were eligible for inclusion. Inclusion criteria included radiologically confirmed chronic subdural hematoma on CT or MRI, patients who underwent surgical evacuation, and availability of complete clinical and radiological data. Exclusion criteria included acute subdural hematoma requiring emergency management, incomplete medical records, and patients managed conservatively.

Data collection

Data were retrospectively collected from medical records and hospital databases using a standardized data extraction form. Demographic variables included age and sex, with sex recorded and analyzed as a demographic variable in the statistical analysis. Clinical data included history of head trauma, comorbidities such as hypertension, diabetes, and renal insufficiency, use of anticoagulant or antiplatelet therapy, and neurological status at admission assessed using the Glasgow Coma Scale. Radiological data were obtained from preoperative CT scans and included hematoma laterality (unilateral or bilateral), hematoma density (hypodense, isodense, hyperdense, or mixed), hematoma thickness in mm, midline shift in mm, and presence of internal septations. Surgical data included type of anesthesia (local or general), surgical technique (burr-hole drainage or craniotomy), use of postoperative subdural drainage, duration of drainage, and presence of postoperative pneumocephalus.

Outcome measure

The primary outcome was recurrence of CSDH, defined as the reappearance of a symptomatic hematoma requiring repeat surgical evacuation during the follow-up period. Patients were followed clinically and radiologically, with postoperative CT scans performed based on clinical symptoms or routine follow-up visits. There was no fixed follow-up duration, reflecting real-world clinical practice. Only clinically significant recurrences requiring reoperation were considered, which helps minimize the impact of variability in follow-up duration.

Statistical analysis

Statistical analysis was performed using SPSS (Statistical Package for the Social Sciences). Continuous variables were expressed as mean ± standard deviation, and categorical variables were expressed as frequencies and percentages. Comparisons between recurrence and non-recurrence groups were performed using Student’s t-test or Mann-Whitney U test for continuous variables, and chi-square test or Fisher’s exact test for categorical variables. Variables with clinical relevance and/or statistical significance in univariate analysis were included in a multivariate logistic regression model to identify independent predictors of recurrence. Results were expressed as odds ratios (OR) with 95% confidence intervals (CI), and a p-value < 0.05 was considered statistically significant.

Development of the predictive score

A predictive score was developed based on independent predictors identified in the multivariate analysis. Each variable was assigned one point, resulting in a total score ranging from 0 to 3. Patients were stratified into risk categories according to their total score.

Ethical considerations

The study was conducted in accordance with the principles of the Declaration of Helsinki. Patient confidentiality was strictly respected, and all data were anonymized. Given the retrospective nature of the study, informed consent was waived.

## Results

Patient characteristics

**Figure 1:**
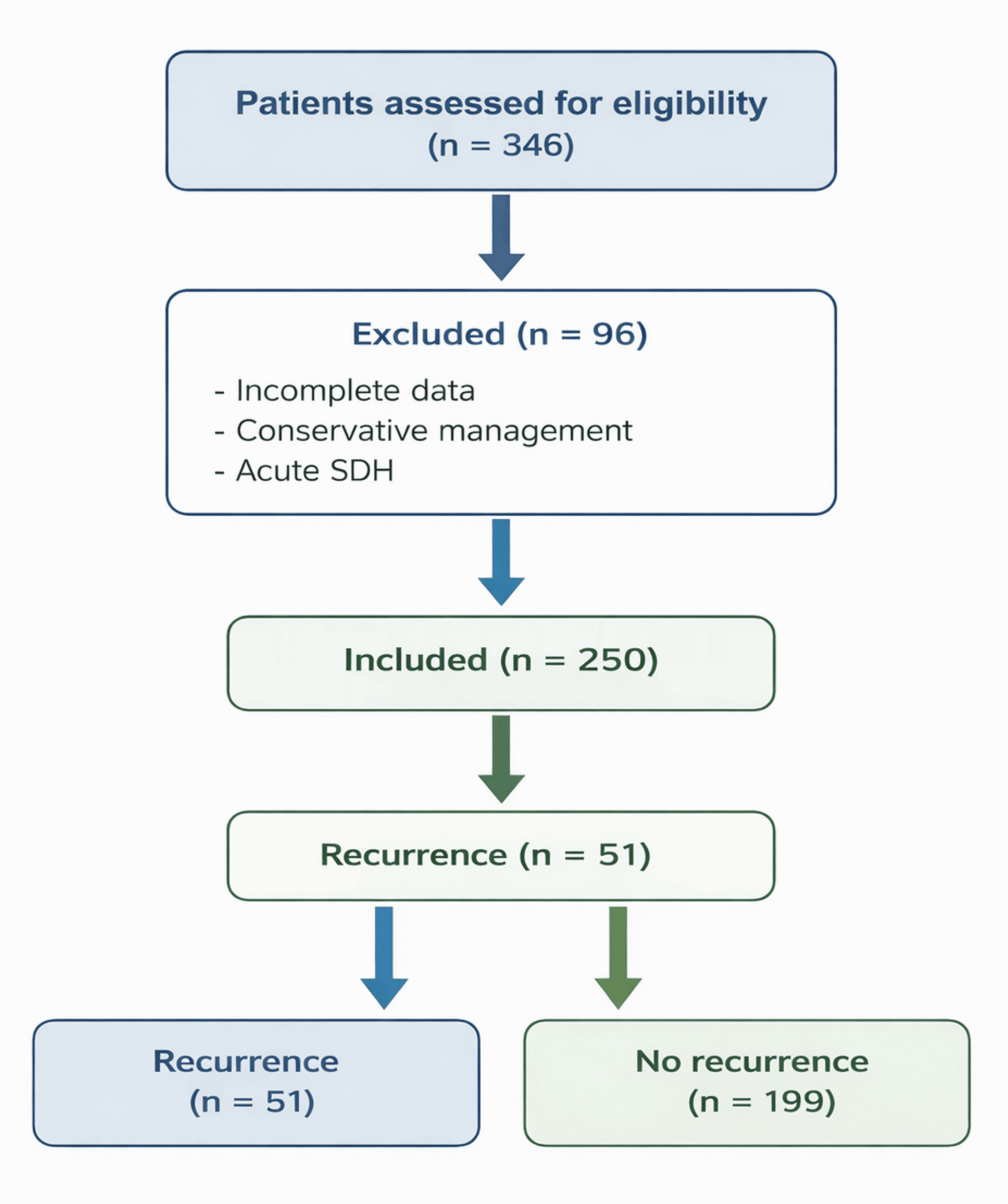
Flowchart of patient selection and study population.

A total of 250 patients undergoing surgical evacuation for chronic subdural hematoma (CSDH) were included in this study. The patient selection process is summarized in Figure 1.

The mean age of the study population was 72 ± 8 years, with a marked male predominance (84%). The overall recurrence rate was 20.4% (51/250 patients). Regarding surgical technique, burr-hole drainage and craniotomy were performed, and no significant association was found between surgical technique and recurrence.

Baseline characteristics

Baseline demographic and clinical characteristics of the study population are summarized in Table 1.

**Table 1:** Baseline characteristics of patients (n=250).

Variable	Value
Age (mean ± SD)	72 ± 8 years
Male sex	210 (84%)
History of trauma	140 (56%)
Hypertension	150 (60%)
Diabetes	50 (20%)
Anticoagulant therapy	110 (44%)
Antiplatelet therapy	40 (16%)

Radiological findings

**Figure 2:**
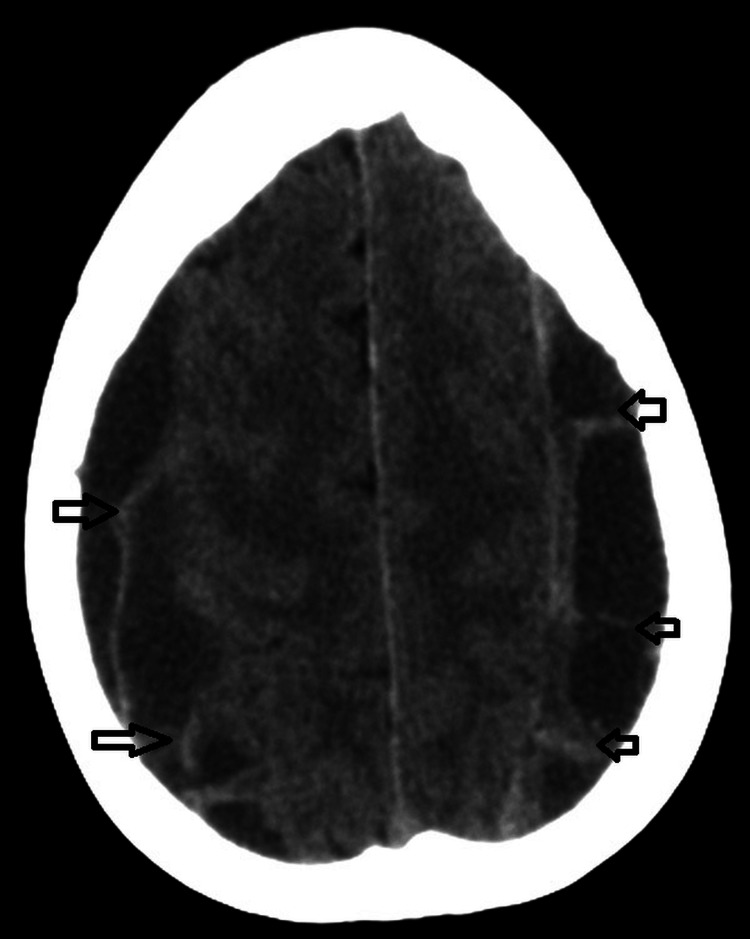
Multiloculated chronic subdural hematoma with internal septations.

**Figure 3:**
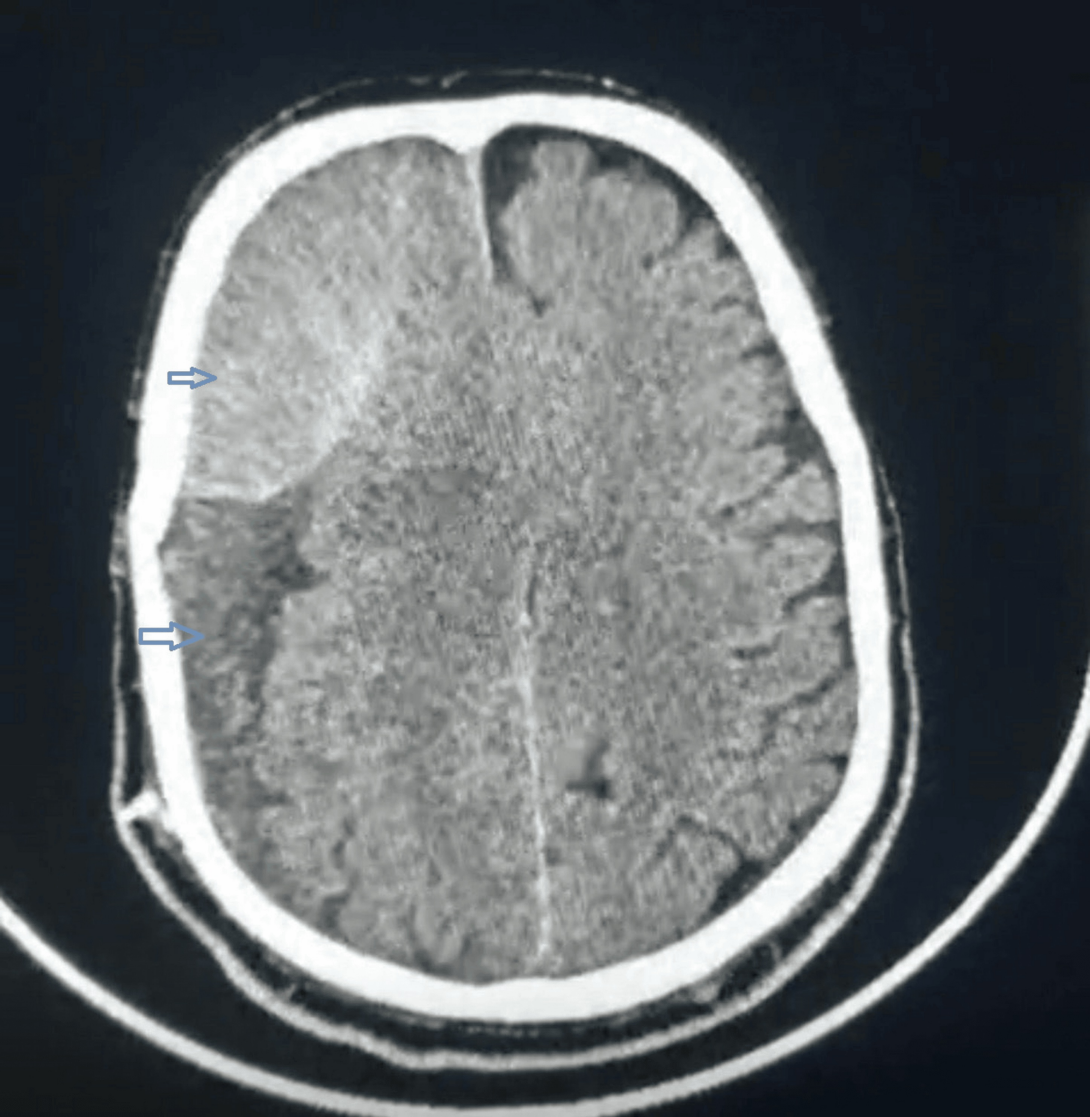
Chronic subdural hematoma with mixed density

**Figure 4:**
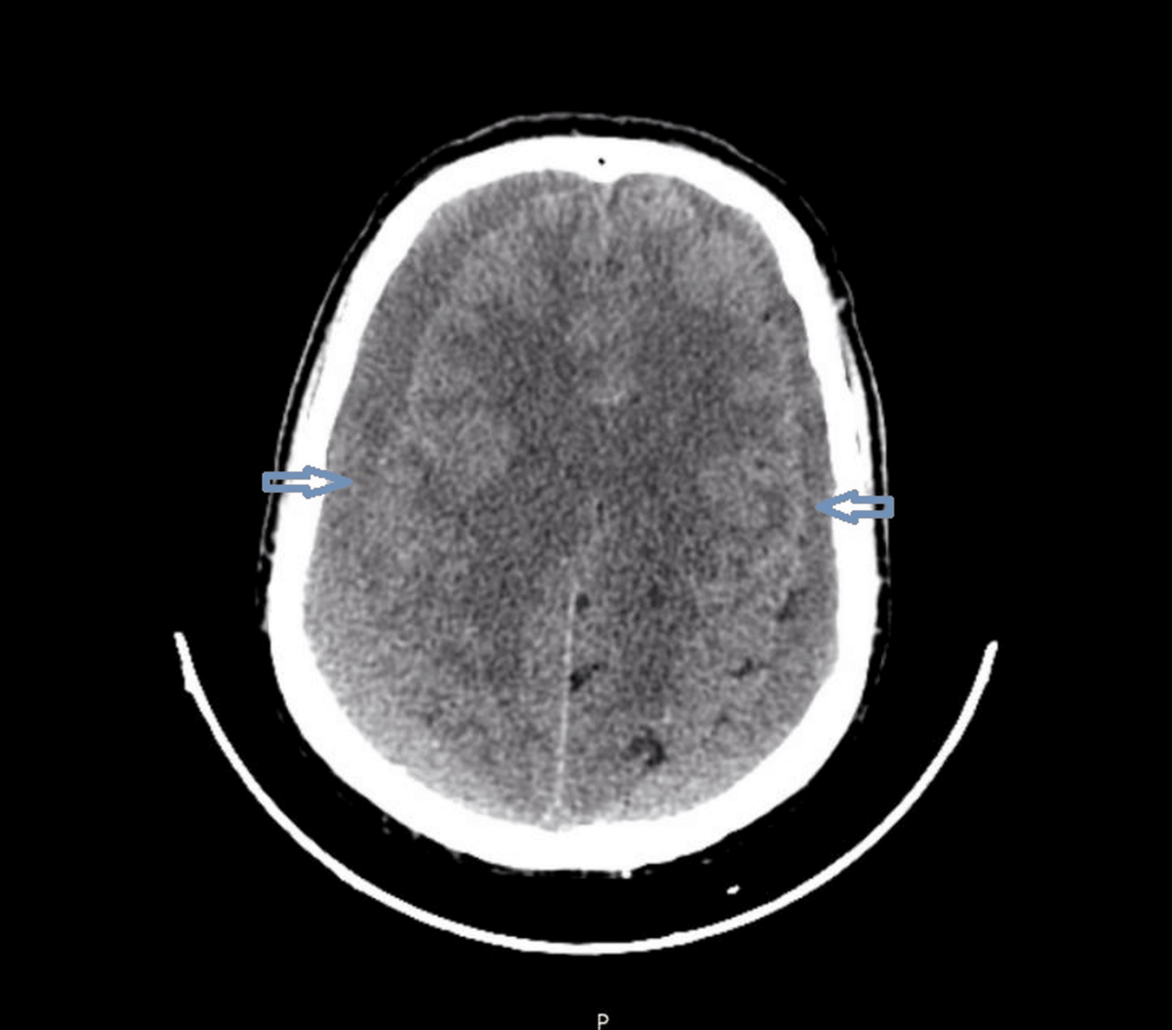
Bilateral chronic subdural hematoma.

**Figure 5:**
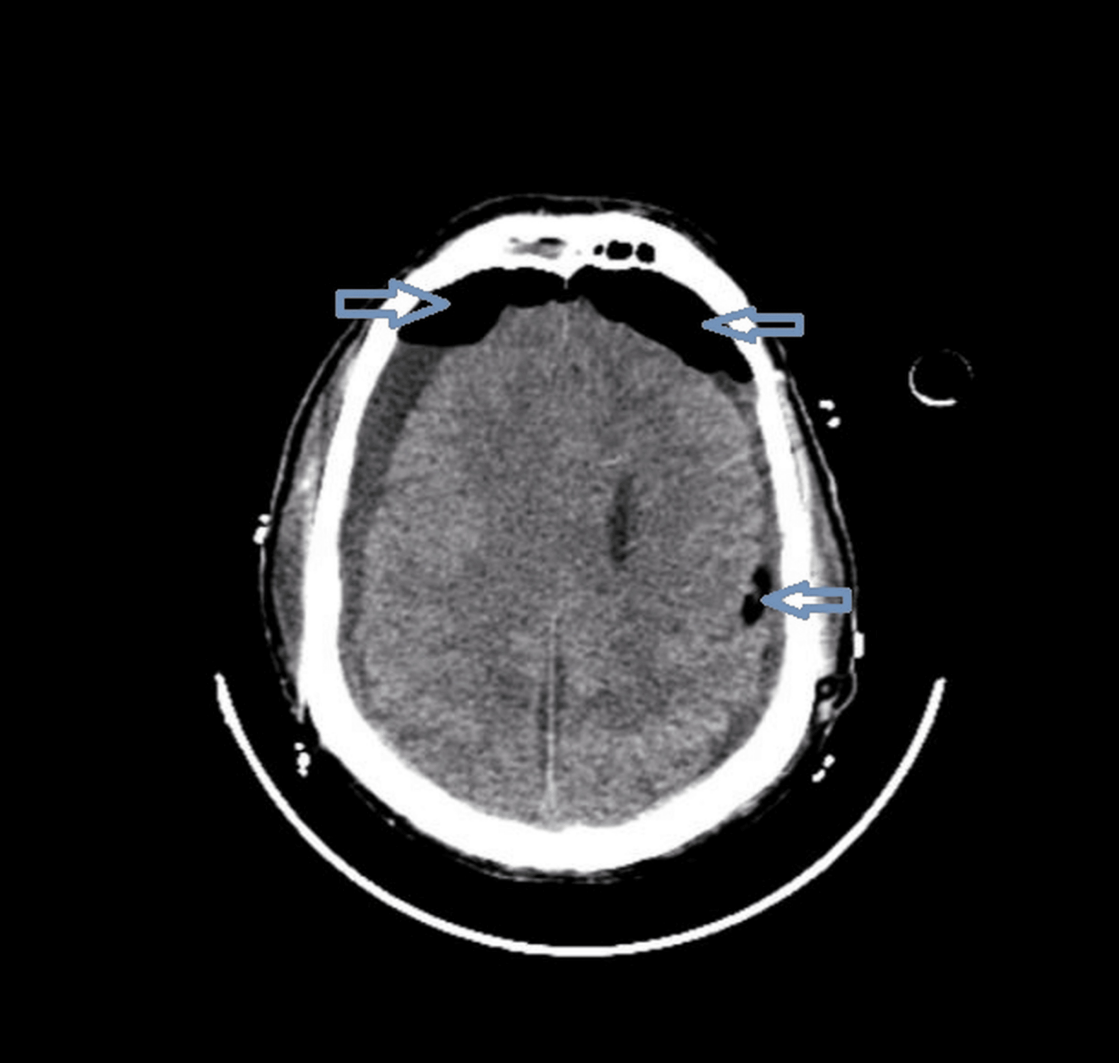
Postoperative pneumocephalus following surgical evacuation.

Radiological analysis showed the presence of several features associated with recurrence, including septations (Figure 2), mixed density (Figure 3), bilateral hematomas (Figure 4), and postoperative pneumocephalus (Figure 5).

No significant differences were observed between recurrence and non-recurrence groups regarding baseline comorbidities such as hypertension and diabetes.

Univariate analysis

Patients were divided into two groups according to recurrence status: the recurrence group (n=51) and the non-recurrence group (n=199). Univariate analysis identified several factors significantly associated with recurrence, including anticoagulant therapy, bilateral hematoma, mixed density, presence of septations, postoperative pneumocephalus, and absence of drainage.

**Table 2:** Univariate analysis of factors associated with recurrence.

Variable	Recurrence (n=51)	No recurrence (n=199)	p-value
Age (years)	74 ± 7	71 ± 8	0.04
Male sex	45 (88%)	165 (83%)	0.30
Anticoagulants	35 (68%)	75 (38%)	0.001
Bilateral hematoma	25 (49%)	60 (30%)	0.02
Mixed density	30 (59%)	70 (35%)	0.01
Septations	32 (63%)	60 (30%)	<0.001
Pneumocephalus	28 (55%)	50 (25%)	<0.001
Drain used	35 (68%)	170 (85%)	0.01

Multivariate analysis

Multivariate logistic regression analysis was performed, including clinically relevant variables. The number of variables included in the model was limited to avoid overfitting, considering the number of recurrence events. Variables included in the multivariate model were age, anticoagulant therapy, bilateral hematoma, mixed density, septations, pneumocephalus, and drainage use. Three independent predictors of recurrence were identified: anticoagulant therapy, presence of septations, and postoperative pneumocephalus.

**Table 3:** Multivariate analysis of recurrence predictors.

Variable	OR	95% CI	p-value
Anticoagulants	2.8	1.5 – 5.2	0.001
Septations	3.5	1.9 – 6.4	<0.001
Pneumocephalus	3.2	1.7 – 5.9	<0.001

Predictive score

Each variable was assigned one point due to comparable effect sizes (odds ratios) observed in the multivariate analysis, allowing for a simple and clinically applicable scoring system. A simple predictive score was developed based on the independent predictors identified in the multivariate model. The variables included anticoagulant therapy, presence of septations, and postoperative pneumocephalus, each assigned one point, resulting in a total score ranging from 0 to 3.

Risk stratification

Recurrence rates increased progressively with higher scores, as shown in Table 4.

**Table 4:** Recurrence risk according to predictive score.

Score	Number of Patients	Recurrence Rate (%)
0	80	5%
1	70	15%
2	60	40%
3	40	70%

**Figure 6:**
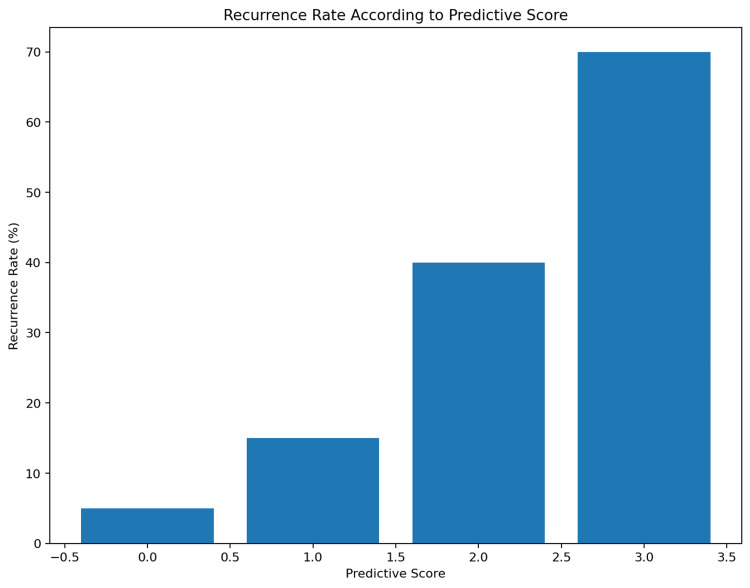
Recurrence rate according to predictive score. A progressive increase in recurrence risk is observed with higher scores, demonstrating the discriminative ability of the proposed scoring system.

Patients with a score ≥2 had a significantly higher risk of recurrence. The recurrence rate according to the predictive score is shown in Figure 6.

Predictive performance

The predictive performance of the score was evaluated using receiver operating characteristic (ROC) curve analysis. The model demonstrated good discriminative ability with an area under the curve (AUC) of approximately 0.80.

## Discussion

Principal findings

In this study including 250 patients undergoing surgical evacuation for chronic subdural hematoma (CSDH), we identified anticoagulant therapy, the presence of septations, and postoperative pneumocephalus as independent predictors of recurrence. The overall recurrence rate of 20.4% observed in our cohort is consistent with previously reported data, confirming that recurrence remains a significant issue in the management of CSDH. Moreover, we developed a simple, clinically applicable predictive score allowing effective stratification of patients according to recurrence risk [1,2,10].

Importantly, recurrence in our study was defined as a clinically significant event requiring reoperation, which represents a robust and objective outcome measure less dependent on follow-up variability.

Recurrence Rate

The recurrence rate observed in our study falls within the range commonly reported in the literature, which varies between 5% and 30%. Despite advances in surgical techniques and perioperative care, recurrence continues to represent a major challenge. Differences in recurrence rates across studies may be explained by variations in patient populations, imaging characteristics, surgical techniques, and duration of follow-up [1-3].

Anticoagulant Therapy

Anticoagulant therapy was identified as a significant independent predictor of recurrence in our study. This association can be explained by impaired hemostasis and a higher propensity for persistent or recurrent bleeding within the subdural space. In addition, anticoagulants may contribute to ongoing microhemorrhages and delay stabilization of the hematoma cavity. However, the impact of anticoagulant therapy remains complex and may depend on perioperative management strategies, including drug interruption and reversal protocols [3-5,11]

Septations (Internal Membranes)

The presence of septations represents one of the strongest predictors of recurrence. Septations reflect an advanced stage of hematoma organization, characterized by neomembrane formation, inflammatory activity, and fragile neovascularization. These structural changes can lead to compartmentalization of the hematoma, preventing complete evacuation and promoting persistence or recurrence. Furthermore, septated hematomas are frequently associated with repeated microbleeding, further increasing the risk of recurrence (Figure 2) [6,7,10].

Mixed Density Hematomas

Mixed-density hematomas are indicative of ongoing bleeding and hematoma instability. These lesions reflect different stages of blood degradation and are often associated with active pathological processes such as fibrinolysis and inflammation. As a result, mixed-density hematomas are associated with a higher likelihood of recurrence compared to homogeneous hematomas (Figure 3) [6,8].

Bilateral Hematomas

Bilateral hematomas have also been associated with an increased risk of recurrence. This may be explained by a larger subdural space and reduced brain re-expansion after surgical evacuation, creating favorable conditions for hematoma reaccumulation. Bilateral involvement may also reflect a more diffuse pathological process affecting the subdural space (Figure 4) [6,9].

Postoperative Pneumocephalus

Postoperative pneumocephalus was identified as an independent predictor of recurrence in our study. The presence of intracranial air may prevent adequate brain re-expansion after surgery, leaving a residual subdural space that facilitates reaccumulation of blood or fluid. Several studies have demonstrated that both the presence and the volume of pneumocephalus are strongly associated with recurrence risk (Figure 5) [5,8,10,12,13].

Clinical implications

The identification of these predictors has important implications for clinical practice. Patients presenting with high-risk features, particularly those included in our predictive score, may benefit from closer postoperative monitoring and tailored management strategies. Preventive measures aimed at reducing pneumocephalus, optimizing surgical techniques, and carefully managing anticoagulant therapy may help reduce recurrence rates. The proposed score should be interpreted with caution as it was derived and tested within the same cohort and lacks external validation.

Strengths of the study

This study presents several strengths, including a relatively large sample size, which increases the statistical reliability of the findings. In addition, the comprehensive evaluation of clinical, radiological, and surgical variables allowed identification of independent predictors of recurrence. Finally, the development of a simple predictive score enhances the clinical applicability of the study and provides a practical tool for routine use.

Limitations

Despite these strengths, several limitations should be acknowledged. The retrospective design may introduce selection and information bias. The single-center nature of the study may limit the generalizability of the findings. The lack of a standardized follow-up duration may have influenced the detection of recurrence. Additionally, the exclusion of patients with incomplete data may have introduced selection bias. Finally, the predictive score was not internally or externally validated and, therefore, requires further validation in independent cohorts. However, by focusing on clinically significant recurrences requiring reoperation, the impact of follow-up variability on outcome assessment is partially mitigated.

Future perspectives

Further research should focus on external validation of the proposed predictive score in larger, multicenter cohorts. The integration of additional imaging features and biological markers may improve predictive accuracy. Emerging therapeutic strategies, such as middle meningeal artery embolization, may also play a role in reducing recurrence rates and should be further investigated.

## Conclusions

Chronic subdural hematoma recurrence remains a significant challenge in neurosurgical practice. In this study, anticoagulant therapy, septations, and postoperative pneumocephalus were identified as significant predictors of clinically relevant recurrence requiring reoperation.

The predictive score developed provides a simple and practical tool for risk stratification. However, given the retrospective design and the absence of a standardized follow-up protocol, these findings should be interpreted with caution.

Nevertheless, this score may offer clinically useful insights for postoperative management in real-world settings. Further validation in larger, multicenter cohorts is required before routine clinical implementation.
